# Expandable-RCNN: toward high-efficiency incremental few-shot object detection

**DOI:** 10.3389/frai.2024.1377337

**Published:** 2024-04-23

**Authors:** Yiting Li, Sichao Tian, Haiyue Zhu, Yeying Jin, Keqing Wang, Jun Ma, Cheng Xiang, Prahlad Vadakkepat

**Affiliations:** ^1^Department of Electrical and Computer Engineering, National University of Singapore, Singapore, Singapore; ^2^Institute of Medicinal Plant Development, Chinese Academy of Medical Sciences and Peking Union Medical College, Beijing, China; ^3^Singapore Institute of Manufacturing Technology, Agency for Science, Technology and Research, Singapore, Singapore; ^4^Robotics and Autonomous Systems Thrust, The Hong Kong University of Science and Technology (Guangzhou), Guangzhou, China; ^5^HKUST Shenzhen-Hong Kong Collaborative Innovation Research Institute, Futian, Shenzhen, China

**Keywords:** object detection, few-shot learning, zero-shot learning, incremental learning, long-tailed recognition

## Abstract

This study aims at addressing the challenging incremental few-shot object detection (iFSOD) problem toward online adaptive detection. iFSOD targets to learn novel categories in a sequential manner, and eventually, the detection is performed on all learned categories. Moreover, only a few training samples are available for all sequential novel classes in these situations. In this study, we propose an efficient yet suitably simple framework, Expandable-RCNN, as a solution for the iFSOD problem, which allows online sequentially adding new classes with zero retraining of the base network. We achieve this by adapting the Faster R-CNN to the few-shot learning scenario with two elegant components to effectively address the overfitting and category bias. First, an IOU-aware weight imprinting strategy is proposed to directly determine the classifier weights for incremental novel classes and the background class, which is with zero training to avoid the notorious overfitting issue in few-shot learning. Second, since the above zero-retraining imprinting approach may lead to undesired category bias in the classifier, we develop a bias correction module for iFSOD, named the group soft-max layer (GSL), that efficiently calibrates the biased prediction of the imprinted classifier to organically improve classification performance for the few-shot classes, preventing catastrophic forgetting. Extensive experiments on MS-COCO show that our method can significantly outperform the state-of-the-art method ONCE by 5.9 points in commonly encountered few-shot classes.

## 1 Introduction

In recent years, the computer vision community shows growing interest in the area of few-shot object detection (FSOD). While existing methods mainly focus on enhancing detection performance of data-rare classes, the critical aspect of computational efficiency is often neglected. In the real-world scenario where detection tasks evolve frequently, novel unseen categories usually come in a consecutive manner, which causes the conventional approaches to suffer from repeatedly model retraining. Moreover, GPU resources are not always accessible or simply too expensive to use in many practical industrial applications, e.g., IoT, robotics, and autonomous vehicles deployed on edge devices (Alabachi et al., 2019). Consequently, this motivates the development of real-world few-shot techniques, which are suitable for online learning and on-site model adaptation. Given the inherently constrained computational resources of mobile devices, it is essential to address efficiency issue in few-shot techniques to fast and accurate model adaptation.

Computation cost for training deep neural networks can be calculated from the following two prospective: computational complexity (FLOPs in each iteration) and adaptation speed (total training iterations). First, computational complexity denotes the computation expense in each iteration during model adaptation. Existing methods computational cost primarily come from three sources: auxiliary networks, trainable parameters, or extra inputs. Second, adaptation speed constitutes a crucial determinant of the adaptation efficiency, which refers to the total training iterations during fine-tuning. From our observation, existing methods (Kang et al., 2019; Yan et al., 2019), which require to fine-tune the feature extraction layers, are more likely to suffer from slow adaptation issue, since there are more trainable parameters. In this article, we study a more challenging detection learning setting, i.e., incremental few-shot object detection (iFSOD) (Perez-Rua et al., 2020). Unlike the conventional FSOD methods, iFSOD requires not only to reduce the time and resource requirement for retraining but also to retain the comparable detection performance. Hence, a desirable solution should contain two important aspects. First, novel classes should be added with rapid adaptation and high accuracy. Second, previous knowledge gained from base classes should be preserved without catastrophic forgetting. According to previous studies (Yang et al., 2020), bounding box regression is category-irrelevant and can be easily generalized to unseen classes without further adaptation. In contrast, bounding-box classification is category-specific, which has to be progressively learned for any new-coming classes. Previous studies employ either a generalizable embedding space (Karlinsky et al., 2019) or feature channel attention (Kang et al., 2019) to facilitate the learning of few-shot classes. However, those approaches are generally unsuited to the iFSOD setting due to the intrinsic need for model fine-tuning. Hence, the objective of this study is to design an incremental few-shot box classifier that can continually expand its capability with new classes in a resource-efficient manner.

To learn from a few samples, imprinting (Qi et al., 2018) is a common approach proposed for modern low-shot classification, which takes the means of normalized embeddings as the class proxies. In this study, we propose to extend the weight imprinting scheme for incremental few-shot detection tasks in order to overcome the overfitting and catastrophic forgetting. Our study reveals that (1) the imprinted foreground-class weights should be IOU-aware, i.e., it should produce higher response to those boxes with larger IOU and (2) the background class should be robust to semantic distribution shift caused by the incrementally added novel classes, which is also IOU-related with foreground classes. To achieve these, we propose an IOU-aware incremental weight imprinting approach to systematically address the incremental weights for both foreground and back- ground classes in iFSOD. Specifically, a prime sample foreground imprinting strategy is proposed to learn the object weights, which pay more attention to the high-quality proposals other than attending all of them equally, and an adaptive background fusion strategy is designed to update the background weight by adaptively fusing background knowledge across different learning steps. As a result, such a zero-training imprinting technique can ideally avoid the overfitting issue in iFSOD, which greatly alleviates the catastrophic forgetting.

However, due to the lack of model fine-tuning, the feature extractor could suffer from category confusion and fail to discriminate between similar classes. As a result, the proxies of novel classes could be drifted away from their desired location and get confused with proxies of retrained base classes, which lead to a biased prediction. Model fine-tuning can be viewed as an explicit way to remit this issue. However, the retraining cost is not affordable in incremental setting as the classification heads always keep extending their size (Singh et al., 2018). To achieve the low computational overhead during model evolvement, instead of fine-tuning the entire detection model with millions of parameters, we explore a middle-ground solution by employing an additional lightweight layer to correct the prediction bias, which is termed as group soft-max layer (GSL). By promoting the correlation between few-shot detection and incremental learning, our approach can rapidly assimilate new categories with only a few annotations without catastrophic forgetting of previous knowledge. Therefore, we term our framework as Expandable Region-based Convolutional Neural Network.

In summary, the main contributions of this study can be listed as three-fold:

- An IOU-aware incremental imprinting strategy is proposed for iFSOD with zero-retraining, which not only promotes the detector classifier to be IOU-aware but also alleviates the background semantic shift issue during the incremental steps.
- The proposed GSL module provides a simple yet effective solution to address the prediction bias issue in iFSOD, where experimental justification is analyzed for validating its effectiveness on bias correction to avoid the catastrophic forgetting.
- The proposed Expandable-RCNN achieves state-of-the-art performance in both iFSOD and normal FSOD settings on standard object detection datasets, e.g., MS- COCO and PASCAL VOC.

## 2 Related works

### 2.1 General object detection

Deep learning-based object detection relies on learning from abundant training data to improve its detection accuracy. Recent detection frameworks can be mainly divided into two groups, i.e., anchor-based and anchor-free. In anchor-based approaches, a set of predefined anchor boxes are predicted by the RPN to roughly cover objects of different sizes and scales. Then, they are further classified into foreground or background patches, and parallelly, refined by an extra regression branch to better fit with ground truth. Representative approaches include Faster Region-based Convolutional Neural Network, FPN, and SSD (Liu et al., 2016; Lin et al., 2017; Saucedo-Dorantes et al., 2020; Zhou et al., 2020). Instead of using anchor box, anchor-free approaches focus on detecting object as key points such as object center or corner, e.g., CenterNet (Zhou et al., 2019) detects objects through predicting object center with spatial size represented by class-specific heatmaps, and CornerNet (Law and Deng, 2018) detects a pair of object corners to form the detection box. The proposed method in this study can be easily plugged into the existing anchor-based frameworks by simply replacing the original classification head with our proposed expandable classifier. Such plug-and-play property makes it practical for incremental and few-shot detection.

### 2.2 Few-shot learning

Few-shot learning is attracting increasing attention due to its realistic applications (Finn et al., 2017; Xue and Wang, 2020). However, most of the existing methods are proposed to address the single image classification problem and are not readily applicable to object detection task. Our method falls within the context of metric learning, and it is mostly related to the previous study on classifier weight imprinting (Qi et al., 2018). We extend its usage from image classification to object detection by explicitly squeezing the prime knowledge from vast proposals.

### 2.3 Few-shot object detection

Most recent few-shot detection approaches are adapted from few-shot learning paradigm; one-shot organ segmentation (OS2) (Yang et al., 2023) leverages a novel support-query interactive embedding module and a self-supervised contrastive learning framework to effectively segment organs from medical images using minimal annotation. FSVOS (Liu et al., 2023) enhances segmentation performance through multi-grained temporal guidance, local and long-term prototype analysis, and a novel loss function. The study mentioned in the reference (Liu et al., 2022b) introduces the Non-Target Region Eliminating (NTRE) network for few-shot semantic segmentation, which effectively identifies and removes background and distracting objects through a novel framework and prototypical contrastive learning. The study mentioned in the reference (Liu et al., 2022a) proposes an Intermediate Prototype Mining Transformer (IPMT) for few-shot semantic segmentation that bridges the category information gap between support and query images through iterative learning. The study mentioned in the reference (Chen et al., 2018) proposes a distillation-based approach with background depression regularization to eliminate the redundant amount of distracting backgrounds. A meta learning-based attention generation network is proposed in the study mentioned in the reference (Kang et al., 2019) to emphasize the category-relevant information by reweighting top-layer feature maps with class-specific channel-wise attention vectors. Sharing the same insight, meta-RCNN (Yan et al., 2019) applies the generated attention to each region proposal instead of the top-layer feature map. TFA (Wang et al., 2020) replaces the original classification head of Faster R-CNN with a cosine classifier to stabilize the adaptation procedure.

However, existing FSOD methods mainly consider the non-incremental learning setting. When new classes are to be added, they generally require retraining the whole framework, which dramatically restricts their scalability into realistic applications such as IoT and robotics, where the access to the expensive computational resource is often prohibitive. To surmount this high-cost model retraining barrier, we consider the more practical incremental few-shot setting, where the proposed Expandable-RCNN is almost training-free for registering novel classes. Moreover, our Expandable-RCNN is also fully compatible with the non-incremental FSOD setting if retraining is allowed.

## 3 Methodology

**Figure 1:**
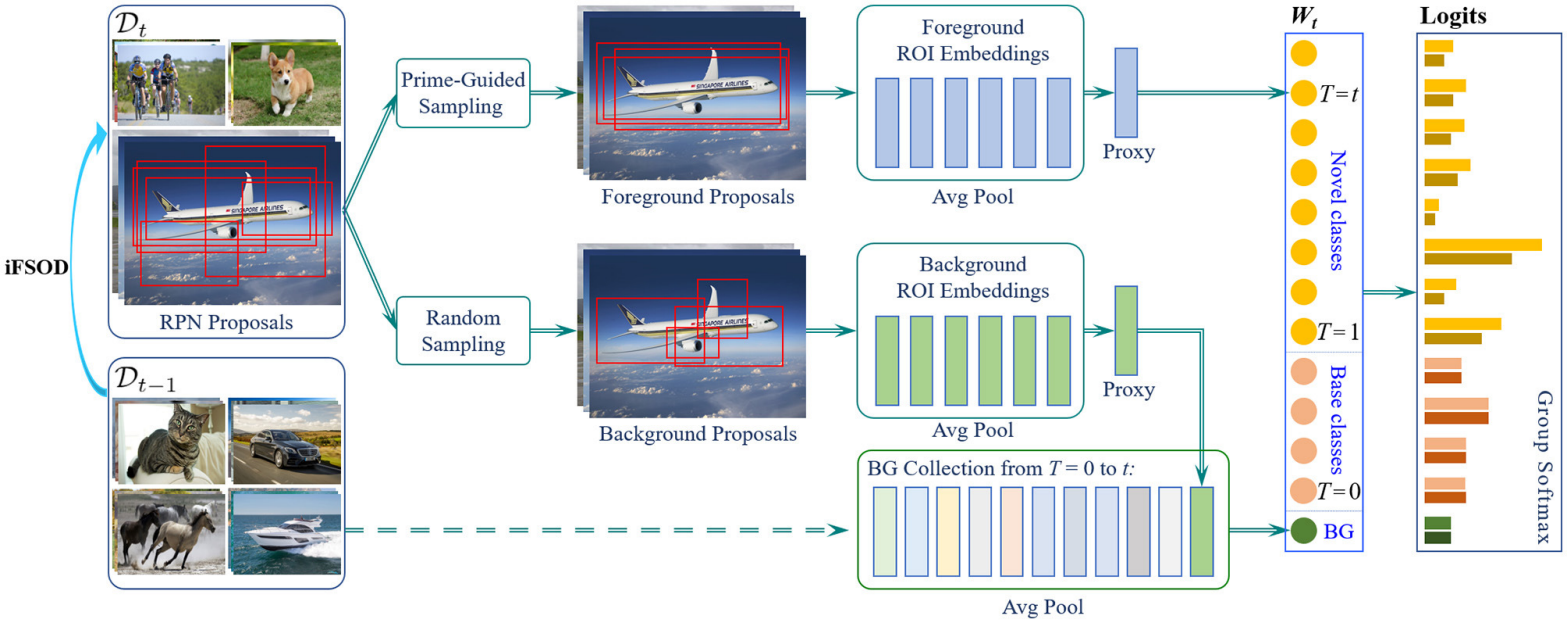
The architecture of our Expandable-RCNN (E-RCNN) framework. When the detector is adapted to a new few-shot task *D*_t_, only the existing box classifier needs to be extended correspondingly. For simplicity, we describe how to update the classification weights for an airplane category. Step 1: Training images of the airplane category are passed through the backbone and RPN to get region proposals (ROIs). Step 2: Prime-sample guided sampling is applied to select a number of confident proposals, according to their IOU with ground-truth boxes. *w*_*obj*_ is formed by summarizing the selected ROI features ϕ_*obj*_ in Eq 4. Step 3: A number of background proposals are randomly sampled to form the background proxy ϕ_*bi*_ for the current class *i*. The background weight *w*_*bg*_ is fused by summarizing all background proxies for experienced classes in a collection *L*_*t*_. Step 4: A bias-correction module (GSL) is learned from an unbiased data distribution to suppress the over-activated logits of new-added classes in order to correct the prediction bias.

The iFSOD problem is formulated as a two-phase learning task in this study. In the first representation learning phase (*t* = 0), a detection model *F*_0_ (·|**W_0, Θ_**) is pretrained on a large set of base classes, where **W**_0_ denotes the pretrained classification weights for base classes and Θ represents all other remaining parameters in the detection model. In the second incremental learning phase (*t* > 0), model updating *F*_t_ (·|***W***_*t*, Θ_) is performed over multiple stages, called learning steps. During the *t*-th learning step, the previous label space *Y*_t − 1_ is expanded with novel classes *C*_*t*_ so that *Y*_*t*_ = *Y*__*t*_−1_ ∪ *C*_*t*_, where *Y*__*t*_−1_ ∩ *C*_*t*_ = ø is assumed for simplicity. To achieve incremental adaptation in Expandable-RCNN, only the new classification head is updated from the previous detector, i.e., ***W***_*t*−1_→*W*_*t*_, while the vast majority of network parameter Θ is frozen. As a result, the proposed Expandable-RCNN is extremely lightweight to expand with novel classes over steps, where its overall pipeline is shown in Figure 1.

### 3.1 IOU-aware incremental weight imprinting

Since each incremental step is few-shot, the classification weights need to be reliably extended without overfitting. In view of the specialties of the detection classification, we propose a novel IOU-aware weight imprinting scheme for systematically addressing the incremental weights for foreground and background classes in iFSOD, which is with zero training step to avoid overfitting.

#### 3.1.1 Prime-sample guided foreground imprinting

Region-based object detection aims at selecting the bounding box which has the maximum overlap with the ground truth from a large pool of candidate proposals. Through NMS, only the bounding box with the largest confidence is preserved while all other nearby proposals are eliminated. Hence, a high-performance object detector should be able to predict higher scores on those positive anchors with higher ground truth IOUs and suppress those only partially overlapped with lower scores. Regarding this, our study reveals that the importance of each proposal depends on how its IOU compares with that of the others that overlap with the ground truth; we argue that the imprinted weights should be more biased toward prime samples with high IOU other than evenly treating all positive proposals. Therefore, we propose the prime-sample guided imprinting strategy to focus more on high IOU proposals instead of treating all proposals equally.

For a feature extractor trained with cosine similarity, the distribution of category proxy could match with the distribution of training samples. If certain samples occur more frequently in the imprinting set, the imprinted weights (proxies) will be closer to those samples in embedding space, which promotes a better classification accuracy on them. Therefore, the resultant classifier is more accurate on the prime samples which have high IOUs. We proposed a biased sampling strategy where the localization accuracy is employed as the measurement of importance to indicate the sample priority. Specifically, taking 1-shot N-way as an example, foreground proposals *p* that overlap with the same object box are divided into four bins according to their IOUs, denoted as *B*_*i*_ in the Equation (1):


(1) $$\begin{array}{l} B_{i}=\left\{p|l_{i}<\text{IOU}\left(p\right)<u_{i}\right\} \end{array}$$

where *i*=1, 2, 3, and 4 and *l*_*i*_ and *u*_*i*_ denote the lower-bound and upper-bound IOU thresholds for *i*-th bin, respectively, and *u*__*i*_−1_ = *l*_*i*_ when *i* > 1. For each IOU interval, proposals are sampled by a different sampling ratio η_*i*_ for class weight imprinting, where η_*i*_ is larger for high-IOU bins and $\sum_{i=1}^{4}\eta_{i}=1$, so the resultant classifier is biased toward high-IOU proposals. Assuming a number of *128* proposals will be selected from each image, there are total *N*_*i*_ proposals in the *i*-th bin, the selected probability.

*P*_*i*_ for each proposal in this bin is calculated by Equation (2):


(2) $$\begin{array}{l} P_{i}=\frac{\eta_{i}\cdot N_{i}}{m}, \end{array}$$

Thus, the sampling function *S*(·) is defined in Equation (3):


(3) $$\begin{array}{l} \varphi_{obj}\sim \;S\left(B_{i},\;P_{i}\right) \end{array}$$

where ϕ_obj_ denotes the sampled foreground ROI features. The class proxy for foreground categories can be calculated by averaging the *L*_2_-normalized vectors of those sampled proposals that belong to the same class:


(4) $$\begin{array}{l} \omega_{obj}=\frac{1}{N_{obj}}\sum \frac{\varphi_{obj}}{{\left||\varphi_{obj}|\right|}_{2}},\;\varphi_{obj}\sim \;\underset{i=1}{\overset{4}{⋃}}S\left(B_{i},\;P_{i}\right), \end{array}$$

where *N*_*obj*_ is the total number of imprinting set. By exploring the generalizable feature representation of prime proposals, ω_*obj*_ can be treated as a robust proxy for novel classes.

#### 3.1.2 Adaptive background fusion

Different from the classical image classification, region-based detectors commonly include a background class for discriminating foreground and background proposals. However, it is observed that the pretrained background weights often fail to generalize beyond the source-domain (base) categories. We conjecture that this is mainly due to the fact that hard negatives can be highly class-specific as they usually cover the most discriminative part of objects. As a result, the semantics associated with the background class always changes when novel classes are incrementally encountered. Consequently, the background parameters used for a certain learning step may not be appropriate for distinguishing hard negatives in the subsequent steps. To tackle this semantic shift issue, we propose to adaptively update the background weights at each incremental step by fully exploring the IOU-aware background information from current and previous learning steps.

Specifically, for learning step *t*, consider its training set *D*_*t*_ = {(*I*_*i*_, *c*_i_)} where *I*_*i*_ denotes the image sample set for a new class $c_{i}\in C^{t}$. For a class *c*_*i*_, a fixed number of *n* background features ${\varphi_{bij}|}_{j=1}^{n}$ are randomly sampled from images *I*ε*I*_*i*_ based on IOU so that its background proxy ϕ_bi_ for class *c*_*i*_ can be approximated by $\varphi_{bi}=\frac{1}{n}\sum_{j=1}^{n}\varphi_{bij}$. To adaptively update the background, ϕ_bi_ will be added to a background collection $L_{t}=L_{t-1}\cap \left\{\;\varphi_{bi}|c_{i}\in C^{t}\right\}\;$ to preserve background proxies for all encounted classes until step *t*, so the adaptive background weights at step *t* can be calculated as follows (Equation 5):


(5) $$\begin{array}{l} \omega_{b}^{t}=\frac{1}{N_{t}}\sum \frac{\varphi_{bi}}{{\left||\varphi_{bi}|\right|}_{2}},\;\varphi_{bi}\in L_{t},\;N_{t}=|L_{t}|, \end{array}$$

Moreover, it is worth noting that the proposed weight imprinting approaches for foreground and background also provide a better starting point than random initialization if fine-tuning is allowed in normal FSOD setting, which results in better detection performance for few-shot categories.

### 3.2 Group softmax layer (GSL)

Although the proposed imprinting approach provides an efficient way for adding new classes, the obtained performance is still away from satisfactory compared with those non-incremental approaches. Due to the lack of model fine-tuning, the pretrained embedding space could be biased toward features that are salient and discriminative among base classes. As a result, the proxies of novel classes could be drifted away from their desired location and get confused with proxies of pre-trained base classes, which causes the proposals from some base classes to be misclassified into novel classes and leads to the key problem of catastrophic forgetting.

**Figure 2:**
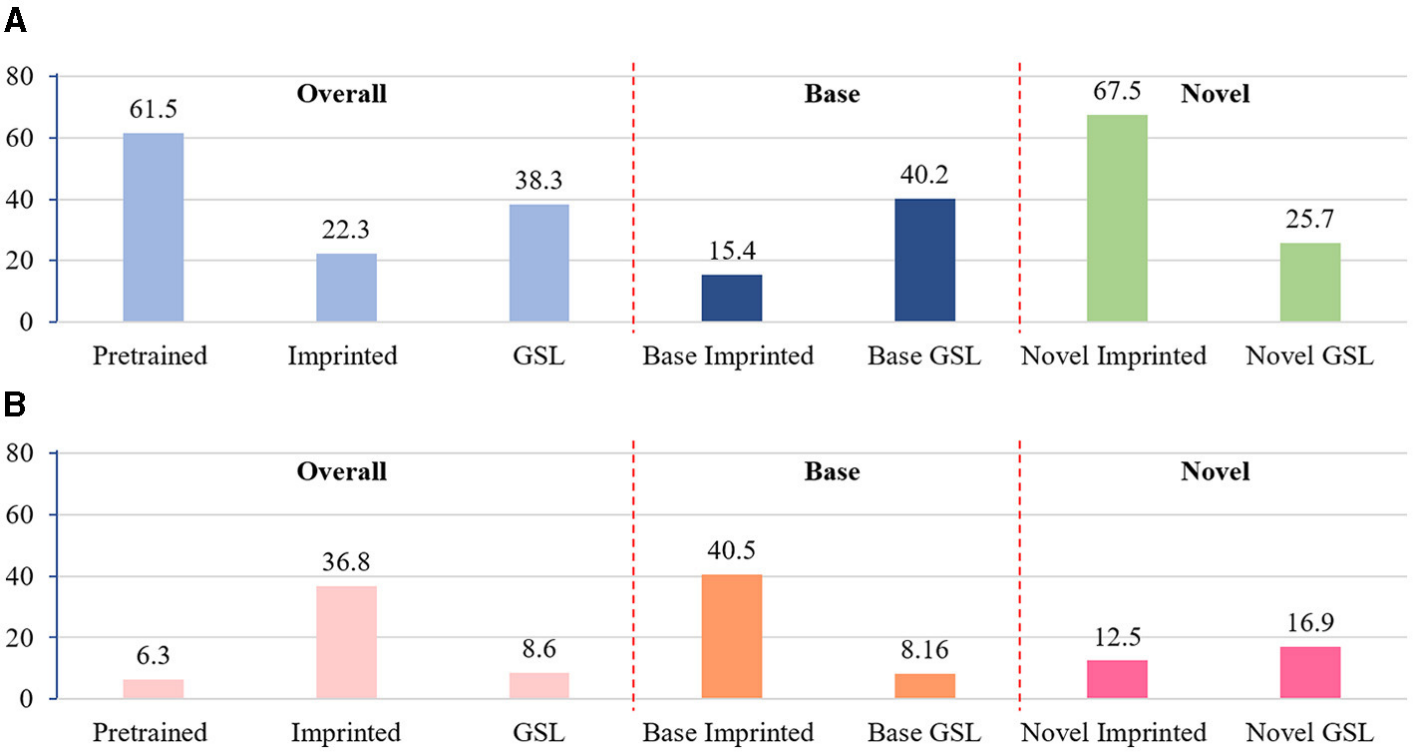
Visualization of the prediction bias. *E*_*acc*_ is calculated based on foreground proposals of base classes, novel classes, and all classes, respectively. “Pretrained” denotes the model pretrained on base set, thus only base classes are presented. “Imprinted” denotes that weights of novel classes are added to the pretrained classifier. “GSL” denotes that model prediction is refined by the proposed GSL model. **(A)** Expected prediction accuracy. **(B)** Expected prediction error.

To validate our hypothesis, extensive experiments are conducted on VOC 2007 test set. Specifically, the prediction confidence for all positive proposals is recorded, and two indicators are designed to reflect the prediction bias: (a) the approximate cosine distance to the proxy of ground truth class-expected prediction accuracy *E*_*acc*_ and (b) the approximate cosine distance to the proxy of hard-negative class-expected prediction error *E*_*err*_. Given a set of region proposals {*p*_*i*_} with their softmax confidence {*c*_*i*_}, the former calculates the average likelihood of making a correct (true-positive) detection, i.e., $E_{acc}=\frac{1}{N}\sum_{i=1}^{N}c_{i}^{u}$, where *u* is the index of ground truth class. The latter evaluates the risk of making a wrong (false-positive) detection, i.e., $E_{err}=\frac{1}{N}\overset{N}{\underset{i=1}{\sum }}c_{i}^{v}$, where *v* is the index which yields the highest response among the rest negative classes. As shown in Figure 2, after extending with new classes, the expected accuracy *E*_*acc*_ for the base classes drops dramatically from 61.5 to 15.4 and the expected error increases significantly from 6.3 to 40.5.

To eliminate such prediction bias, a bias correction module is proposed and incorporated with our iFSOD framework. Concretely, the bias correction module is required to be lightweight with only a few parameters in order to enable fast model updating and overfitting avoiding. We first divide the output logits into three groups, i.e., base classes *C*_*bs*_ (*t* = 0), novel classes *C*_*nv*_ (*t* > 0), and background classes *C*_*bg*_. As shown in Equation (6), the proposed bias diminishing approach is built on top of the last fully connected layer to perform a linear transformation, which is applied to correct the prediction bias on the output logits for the novel classes, while output logits of the other two groups (base and background) are kept to be unchanged.


(6) $$q=\left\{ \begin{array}{l} s_{k}+b_{bs},\;k\in C_{bs}\\ \alpha_{nv}s_{k}+\beta_{nv}+b_{nv}\;,\;k\in C_{nv}\\ s_{k}+b_{bg},\;k\in C_{bg} \end{array}\right.$$

where α_*nv*_ and β_*nv*_ denote two tunable parameters, *s*_*k*_ denotes the classification logit of a specific class, and *b*_*bs*_ denotes a learnable bias parameter. The final prediction is calculated in Equation (7):


(7) $$\begin{array}{l} p=Soft\text{max}\left(q\right), \end{array}$$

To better learn the prediction bias, training samples for GSL should be unseen to the detector. Specifically, its training set contains equal number of samples for all existing classes. Samples selected for base classes are excluded from detector pretraining, while samples for novel classes are the same as those used for weight imprinting. During each learning step *t*, the GSL is re-initialized and learned jointly with a standard softmax cross-entropy loss.

The effectiveness of GSL is presented in Figure 2, which shows the significant *E*_*err*_ reduction on base classes with the correction of over-activated *E*_acc_ on novel classes. With the help of an unbiased validation set, GSL enables a biased classifier to calibrate its prediction and correct its bias, which provides an efficient way to reduce category confusion caused by the lack of fine-tuning. However, one may wonder if the reduced *E*_acc_ on novel classes could lead to any performance degradation. We argue that the absolute value of *E*_acc_ is not always related to the final detection performance as the non-maximum-suppression is performed on proposals of each class independently. In contrast, *E*_err_ is more meaningful as it is often inversely proportional to the total amount of false positives. As presented in Figure 2, the overall expected error *E*_err_ is significantly reduced after introducing GSL, thus the detector is expected to perform much better after more false positives are removed.

## 4 Experiments

### 4.1 Experiment setting on MS-COCO

For the first same-domain evaluation, we train our model on the union of the 80K train set and 35K trainval set of MS-COCO, and test on the 5K minival set. For the total 80 categories, 20 categories that are overlapped with Pascal VOC are selected as novel classes, and the remaining 60 classes serve as base classes. It is worth noting that base set images may also contain instances from novel classes. However, we do not provide any annotations for novel class instances during pretraining. Upon the arrival of each novel class, the model can only access the annotation of k instances, where *k* = {1, 5, 10, 30} for all experiments. For the cross-domain evaluation from MS-COCO to VOC, the model is first trained on MS-COCO and then evaluated on VOC2007 test set. Different from the first experiment that focuses on evaluating cross-category model generalization, this setup is to further appraise the cross-domain generalization ability.

### 4.2 Experiment setting on Pascal-VOC

For the Pascal VOC dataset, we train our model on the union of 07 and 12 train/validation set and evaluate on 07 test set. In particular, we consider the following split of base and novel class: bird, bus, cow, bike, and sofa/the other. During training, the model can only access to k instances for each few-shot class, where we set *k* = {1, 2, 3, 5, 10}.

### 4.3 Implementation details

We use Faster R-CNN as our base detector, and an ImageNet pretrained Resnet-50 (He et al., 2016) is employed as the backbone. For the representation learning phase (*t* = 0), a detector model is trained with inner product similarity in the first 10 epochs using SGD with a minibatch size of 16, learning rate of 0.02, momentum of 0.9, and weight decay of 0.0001. Then, we freeze the feature extractor and RPN while only fine-tune the *conv5* layer and detection heads with cosine-similarity for another two epochs using a smaller learning rate of 0.006. In the second incremental learning phase (*t* > 0), to adapt the existing detector with new classes, we select *m* = 40 foreground proposals and 32 background proposals from each few-shot training sample for weight imprinting. The foreground IOU interval is split into four bins {0.5–0.6, 0.6–0.7, 0.7–0.8, and 0.8–1.0} in (1). The sampling ratio {η_*i*_} for each bin is set to {0.1, 0.2, 0.3, and 0.4}. For the learning of GSL model, we initialize the temperature parameter α_*nv*_ to be 1 and the bias parameter β_*nv*_ to be 0. GSL is trained using SGD with a learning rate of 0.01 and momentum of 0.9. A fixed number of 32 foregrounds and 96 backgrounds are sampled from the top 300 proposals for each training image. Each minibatch is constructed with 12 images. All experiments are implemented on PyTorch 1.0 and trained with 4 RTX 2080 Ti GPU.

### 4.4 Incremental few-shot object detection

#### 4.4.1 Experimental setup

To evaluate our approach, we follow the common practice of *k*-shot but with the key difference that only the GSL model is learned while the other part of the detection model is frozen. In particular, our approach is evaluated on the commonly used object detection benchmarks, Pascal VOC (Everingham et al., 2010) and MS-COCO (Lin et al., 2014), following the same experiment setting with meta-RCNN (Yan et al., 2019).

For the first same domain evaluation, we train our model on the union of the 80K train set and 35K trainval set of MS-COCO and test on the 5K minival set. For the total 80 categories, 20 categories that are overlapped with Pascal VOC are selected as novel classes, and the remaining 60 classes serve as base classes. It is worth noting that base set images may also contain instances from novel classes. However, we do not provide any annotations for novel class instances during pretraining. Upon the arrival of each novel class, the model can only access the annotation of k instances, where *k* = {1, 5, 10, 30} for all experiments. For the cross-domain evaluation from MS-COCO to VOC, the model is first trained on MS-COCO and then evaluated on VOC2007 test set. Different from the first experiment that focuses on evaluating cross-category model generalization, this setup is further to appraise the cross-domain generalization ability.

#### 4.4.2 Results on MS-COCO

As shown in Table 1, on the proposed few-shot detection benchmark, we have compared our approach with three baselines. For the first baseline denoted as “Feature-Reweight” (Kang et al., 2019), it is a meta-learning-based approach which is originally designed for non-incremental learning and is adapted to the incremental setting (Perez-Rua et al., 2020). The second baseline is the state-of-the-art approach ONCE (Perez-Rua et al., 2020), which is also the only incremental few-shot detection benchmark available to date. The third baseline is a simplified version of our framework, where only the proposed imprinting process is preserved, denoted as “E-RCNN w/o GSL”. Regarding the results, we have several observations. (1) The accuracy of the existing meta learning approaches is still far away from satisfaction. Although they can be further improved after fine-tuning the meta model, the episodic learning scheme is memory inefficient when the number of classes increases, which makes them not feasible for real-world iFSOD scenarios. (2) Compared with the baseline “E-RCNN w/o GSL”, the GSL model can bring significant AP (Average Precision) improvement of 0.7 points on base classes, which naturally solves the issue of catastrophic forgetting. (3) Our method achieves the best performance in all different data splits and different numbers of training shots. The improvements, up to 6.8 points in mAP (mean Average Precision), indicate the effectiveness of our approach. (4) The proposed imprinting approach outperforms the original imprinting approach by 1.4 points under the 10-shot scenario, which indicates blindly making use of all the positive anchors can lead to degraded performance. We conjecture this because the distribution of ROIs generated by the RPN is highly skewed to low IOU levels (Pang et al., 2019). Random sampling may result in the imprinted weights to be biased toward low IOU level and thus fails to recognize high IOU anchors that are more crucial for improving detection performance.

**Table 1:** Comparison with iFSOD baselines on MS-COCO.

**Methods**	**Novel AP**			**Base AP**		
	**1-shot**	**5-shot**	**10-shot**	**1-shot**	**5-shot**	**10-shot**
Feature-reweight; Kang et al. (2019)	0.1	0.8	1.5	2.5	3.3	3.4
ONCE; Perez-Rua et al. (2020)	0.7	1.0	1.2	17.3	16.9	19.7
Meta-RCNN; Yan et al. (2019)	1.6	2.3	4.2	21.5	20.3	20.1
Imprinting; Qi et al. (2018)	3.0	4.3	6.6	23.6	24.9	24.6
EWC; Kirkpatrick et al. (2017)	3.1	4.0	4.6	11.6	14.9	15.8
IMM; Lee et al. (2017)	3.3	4.6	6.5	23.4	22.9	24.2
E-RCNN w/o GSL	3.5	5.8	7.8	27.3	27.4	28.0
E-RCNN	**3.7**	**6.1**	**8.0**	**28.5**	**28.5**	**28.7**

Bold values indicate mAP (mean Average Precision).

#### 4.4.3 Results on MS-COCO to Pascal VOC

We then evaluated the proposed method in a cross-dataset setting, where the model is first trained on MS-COCO and evaluated on PASCAL VOC2007 test set. The results show that our approach outperforms the state-of-the-art ONCE by 10.1 points under 10-shot case (2.6 vs. 12.7), which confirms the generalization advantages of our method when transfers to a test domain different from the training one.

#### 4.4.4 Results on Pascal VOC

We provide the AP50 performance on VOC 07 test set, as shown in Table 2. Our methods consistently outperform meta-learning approaches by approximately 10–20 points in fewer shots and achieve comparable results with the state-of-the-art non-incremental approach TFA, though our model does not fine-tune. In all experiments, the performance gain of GSL is significant and robust for base classes, i.e., up to 5 points improvement in AP50, which validates its remarkable performance in distinguishing catastrophic forgetting caused by prediction bias. However, when the total shots get fewer, the performance gain on novel classes becomes lower simultaneously; since under the extremely low data diversity, the model may fail to capture the intra-class variation of the category (Hariharan and Girshick, 2017).

**Table 2:** Incremental learning results on Pascal VOC.

**Methods**	**Novel AP**					**Base AP**				
	**1-shot**	**2-shot**	**3-shot**	**5-shot**	**10-shot**	**1-shot**	**2-shot**	**3-shot**	**5-shot**	**10-shot**
Feature-reweight; Kang et al. (2019)	14.8	15.5	26.7	33.9	47.2	53.2	55.9	55.6	57.0	59.5
ONCE; Perez-Rua et al. (2020)	15.3	18.7	30.0	39.5	46.8	54.2	58.5	57.9	56.1	62.8
Meta-RCNN; Yan et al. (2019)	19.9	25.5	35.0	45.7	51.5	56.1	59.3	57.3	61.6	63.8
Imprinting; Qi et al. (2018)	36.3	38.2	40.7	45.1	45.6	63.8	64.0	65.6	65.9	66.0
EWC; Kirkpatrick et al. (2017)	19.8	20.5	21.2	22.3	24.0	42.8	43.6	42.1	44.7	45.9
IMM; Lee et al. (2017)	22.3	23.7	25.9	27.8	30.4	50.3	52.5	51.7	53.8	52.4
E-RCNN w/o GSL	**40.5**	**42.6**	43.3	46.3	47.7	64.9	65.1	65.7	65.0	64.9
E-RCNN	40.0	42.3	**43.5**	**48.1**	**49.8**	**67.2**	**67.5**	**68.3**	**68.3**	**68.6**

Bold values indicate mAP (mean Average Precision).

### 4.5 Efficiency analysis

We evaluate the efficiency of various baselines during model adaption. It is important to note that the adaption efficiency depends on both computational complexity and adaptation speed. First, we compute the FLOPs for each iteration and average the results among different iterations to get the final computational complexity. We implement this through using the open-source library “DeepSpeed”. Next, we measure adaptation speed by counting the total training iterations for the adaptation process. Furthermore, we also report the actual memory cost during few-shot adaptation to directly reflect the memory efficiency of different methods. In particular, we utilize three metrics to measure the computational efficiency of the different baselines.

Computational complexity: calculating the computational cost of training a deep neural network in FLOPs involves considering both the inference step and the backward gradient step. In this study, we use Faster R-CNN with Resnet-50 as the back-bone, and we calculate computational complexity based on FLOPs in a single training iteration, with a fixed image size of 1,000 × 800. In particular, model training is based on the top 256 ROI extracted by RPN. As shown in Table 3, our method could achieve the best trade-off between computational complexity and detection performance. As we can observe, efficiency issues remain a major concern for meta-learning methods, since the addition of a support branch considerably leads to larger computational cost. The naive imprinting method achieves slightly lower computational complexity than our approach, but it yields worse detection performance. We believe that this is caused by underfitting due to the lack of necessary fine-tuning of model parameters. On the contrary, the proposed GLS model can effectively alleviate this issue and generate unbiased prediction for novel classes, with negligible computation cost.

**Table 3:** Results of computation efficiency on MS-COCO dataset.

**Methods**	**FLOPs (G)**	**Iterations**	**Memory (Gb)**	**mAP**
Feature-Reweight; Kang et al. (2019)	980.2	1.5*k*	4.04	1.5
ONCE; Perez-Rua et al. (2020)	1,679.1	2.0*k*	7.81	1.2
Meta-RCNN; Yan et al. (2019)	1,280.0	1.8*k*	6.35	4.2
Imprinting; Qi et al. (2018)	270.3	0.2*k*	1.02	6.6
E-RCNN w/o GSL	270.3	0.2*k*	1.05	7.8
E-RCNN	270.9	0.2*k*	1.06	8.0

Adaptation speed: We define adaptation speed as the total training iterations during fine-tuning. To avoid overfitting, we employ an early stopping strategy during the few- shot adaptation. Meta-learning methods require to fine-tune the ROI feature extractor that contains millions of parameters, which leads to poor adaptation speed. From the result, our method adapts significantly fast, achieving seven times adaptation speed boost compared with the meta-learning methods.

Memory cost: in addition to other performance metrics, memory cost serves as an important metric of overall computational efficiency during the backward-propagation process, as it is directly tied to the number of trainable parameters in the model. A com- parison presented in table reveals that our proposed method requires significantly less GPU memory than alternative meta-learning approaches during model adaptation. This can be attributed to our concise network design, where the proposed GSL model only consists of a few trainable parameters, resulting in less memory overheads. For instance, our method consumes a mere 16.7% of the memory required by meta- RCNN, demonstrating its superior efficiency in terms of memory utilization. Comparing with the naive imprinting baseline, the proposed IOU-aware imprinting additionally consumes 4% memory cost, with a relative improvement of 21.2% percent in detection accuracy, which indicates that our method achieves a better efficiency performance trade-off.

### 4.6 Ablation study

#### 4.6.1 Extensive experiments on GSL model

**Figure 3:**
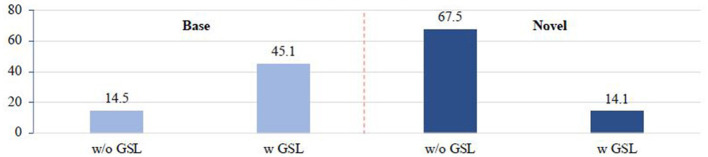
Averaged activation strength on base and novel classes: “w/o GSL” denotes only the imprinted classifier is used for evaluation; “w GSL” denotes that the final prediction is further refined by the proposed GSL model.

We first analyze the averaged activation on base and novel classes, respectively. In particular, we collect the softmax distributions from all foreground proposals. We average all the softmax distributions to get the mean distribution. The averaged activation strength on base classes is calculated by summing all the scores that belong to base classes. Calculation for novel classes can be done in the same way. In Figure 3, before applying GSL model, the imprinted classifier presents much larger activation strength on novel classes than base classes, which indicates that the logits of novel classes is over-activated. Regarding this issue, GSL can inhibit the over-activated logits of novel classes with two bias parameters, which successfully eliminates the above prediction bias.

**Figure 4:**
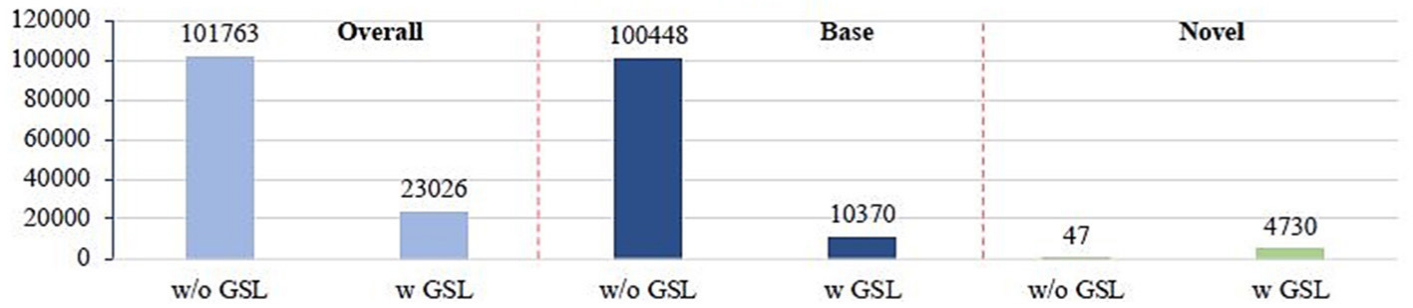
Comparison of number of false positives.

To validate the performance of the proposed GSL model, we evaluate an imprinted Faster R-CNN on Pascal VOC 2007 test set and count the total number of misclassified proposals before and after applying GSL. In particular, we collect all the foreground proposals from the test set. According to the classification results, we divide them into two groups. (1) All the proposals that are misclassified into a wrong class. (2) Proposals belong to base classes but are misclassified into one of the novel classes. (3) Proposals belong to novel classes but are misclassified into one of the base classes. Regarding the results shown in Figure 4, we have several observations, (1) Over 90% of the misclassified proposals are from base classes but are falsely classified into novel classes, which is mainly due to the biased prediction caused by the overactivated novel class logits. (2) GSL can effectively inhibit the over-activated logits of novel classes, thus significantly reduce over 90% percent of the misclassified base class proposal (from 100,448 to 10,370), as well as the overall mistakes (from 101,763 to 23,026). (3) As a side effect, logits of novel classes may be overinhibited, which causes a small amount of novel-class proposals to be misclassified into base classes. However, this effect is negligible since the overall mistake is reduced, which indicates that smaller category confusion is achieved after applying GSL. This can be further validated by the fact that final detection performance on novel classes is significantly improved (more than 2 points under AP50).

#### 4.6.2 Ablation on each component

To evaluate the importance of each component, we gradually add prime-sample guided foreground imprinting (FG-IMP), adaptive background fusion (BG-IMP), and group softmax layer (GSL) to our framework. The results are shown in Table 4. First, without background fusion, background weight is always set to be $\omega_{t=0}^{bg}$ for all learning steps. The detection performance decreases from 8.0 (FG-IMP + BG-IMP) to 7.3 (FG- IMP only), which reveals that semantic distribution shift is a key issue when fine-tuning is not allowed. Second, the GSL model can significantly improve the detection performance on base classes from 28 to 28.7, validating the effectiveness of this simple method in preventing catastrophic forgetting. As we can observe, different components of our framework are simple yet effective. They can cooperatively address incremental few-shot detection in a unified manner.

**Table 4:** Ablation study of each component.

**Shot**	**Methods**	**Novel**	**Base**
10	FG-IMP	7.3	27.9
	FG-IMP + BG-IMP	7.8	28.0
	FG-IMP + BG-IMP + GSL	**8.0**	**28.7**

Bold values indicate mAP (mean Average Precision).

#### 4.6.3 Ablation on different sampling strategies

To study the effects of different sampling strategies, random sampling, IOU-balanced sampling, and prime-sample guided sampling are applied on positive samples, respectively. As shown in Table 5, the proposed imprinting approach outperforms randomly sampling by 1.4 points, which indicates blindly making use of low quality proposals may lead to noisy weights. The distribution of ROIs generated by the RPN is highly skewed to low IOU levels (Pang et al., 2019). Randomly sampling may result in the imprinted weights to be biased toward low IOU level, thus performs poorly in recognizing high IOU anchors. IOU-balanced sampling provides only small improvements (0.4 points higher AP) compared with random sampling, which reveals that assimilating balanced information from each IOU interval is not necessary. In contrast, our method achieves the best performance, since it can enforce the classifier to be more accurate on samples with high IOUs, which are also more important for the overall detection performance (Cao et al., 2020).

**Table 5:** Ablation study on sampling strategies.

**Shot**	**Methods**	**Novel**	**Base**
10	Random sampling	6.6	27.9
	IOU-balanced sampling	7.0	28.5
	Ours	**8.0**	**28.7**

Bold values indicate mAP (mean Average Precision).

Then, we evaluate the effectiveness of prime-sample guided sampling with different hyper-parameters *k*, which denote the number of intervals. Experiments in Table 6 show that the results are very close to each other when the parameter *k* is set as 4, 6, and 8. Therefore, the number of sampling intervals is not very crucial in our prime-guided sampling.

**Table 6:** Ablation study on hyper-parameters *k*.

**Shot**	***k***	**Novel**	**Base**
10	4	7.5	27.8
	6	7.9	28.0
	8	**8.0**	**28.7**

Bold values indicate mAP (mean Average Precision).

#### 4.6.4 Ablation on architecture of GSL

We also study the impact of different architecture designs for the GSL model. We compare four options, where the bias-correction transformation is applied to (1) base group, (2) novel group, (3) base and novel group, and (4) all the three groups. The results are shown in Table 7. Although including base and background class provide slightly better performance on base classes, performance on the new few-shot classes is degraded significantly. Thus, the bias-correction technique is more suited to be applied to novel classes only.

**Table 7:** Ablation study on GSL model.

**Shot**	**Methods**	**Novel**	**Base**
10	None	6.8	28.0
	Base	7.4	28.4
	Novel (ours)	**8.0**	28.7
	Base+Novel	7.5	**28.9**
	All	7.5	**28.9**

Bold values indicate mAP (mean Average Precision).

## 5 Conclusion

In this study, we propose an essentially generic learning scheme Expandable-RCNN for incremental few-shot detection. First, the proposed IOU-aware imprinting approach can not only effectively assimilate a new few-shot detection task by focusing on prime samples but also naturally solve the issue of semantic shift by accumulating background knowledge from history. Second, the proposed lightweight GSL module can efficiently calibrate the biased prediction with only a few parameters, which provides a better trade-off between efficiency and accuracy. To sum up, our approach is simple yet effective and performs remarkably in few-shot object detection. The effectiveness is validated by extensive experiments, where our approach yields state-of-the-art performance on MS-COCO and significantly outperforms the baseline algorithm by 5.9 points.

## Data availability statement

The original contributions presented in the study are included in the article/supplementary material, further inquiries can be directed to the corresponding author.

## Author contributions

YL: Conceptualization, Methodology, Writing – original draft, Project administration, Validation. ST: Conceptualization, Methodology, Writing – original draft, Data curation, Writing – review & editing. HZ: Investigation, Methodology, Project administration, Software, Writing – original draft. YJ: Funding acquisition, Resources, Writing – original draft. KW: Formal analysis, Project administration, Validation, Writing – original draft. JM: Supervision, Writing – review & editing. CX: Supervision, Writing – review & editing. PV: Supervision, Writing – review & editing.
